# Supplementary material for: On the Role of ROS and Glutathione in the Mode of Action Underlying Nrf2 Activation by the Hydroxyanthraquinone Purpurin
Source: Antioxidants (Basel). 2023 Aug 2;12(8):1544. doi: 10.3390/antiox12081544 (PMC10451334; doi:10.3390/antiox12081544)
Supplement: Supplementary file 1 [file antioxidants-12-01544-s001.zip › Supplementary Materials.pdf]

Supplementary file belonging to:

On the role of ROS and glutathione in the mode of action underlying Nrf2 activation by the hydroxyanthraquinone purpurin

Qiuhui Ren, Wouter Bakker, Sebastiaan Wesseling, Hans Bouwmeester, Ivonne M.C.M. Rietjens

Division of Toxicology, Wageningen University and Research, Stippeneng 4, 6708 WE, Wageningen, The Netherlands

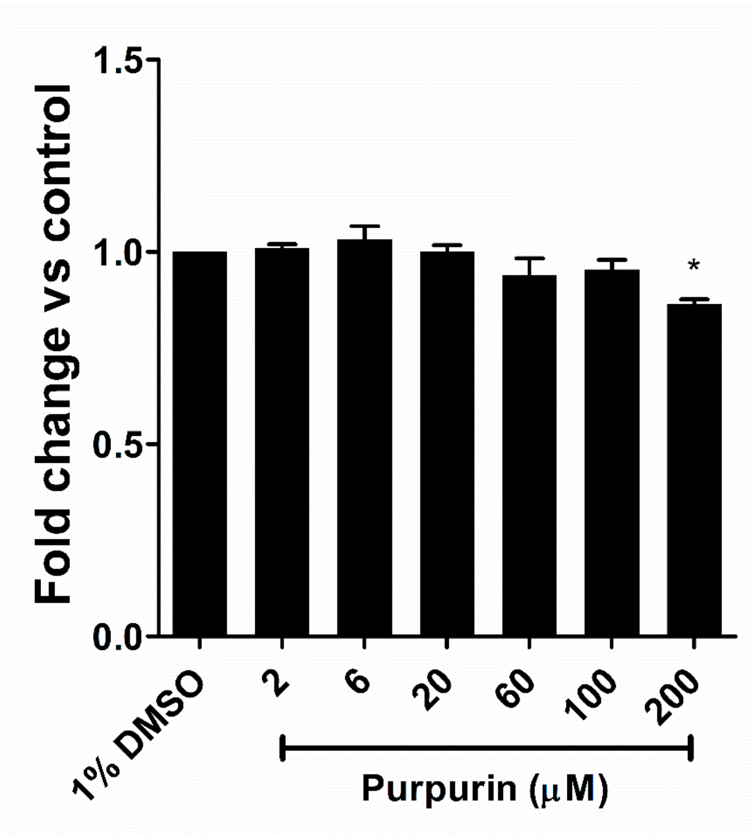

Figure S1. Quenching effect of purpurin on fluorescence intensity in a cell-free DCF-DA assay. The fluorescent molecule, DCF was directly incubated with purpurin for 4 hours. Data are shown as mean fold increase compared to solvent control ± SEM of 3 replicates (*, $p<0.05$; one-way ANOVA analysis with post-hoc Tukey test).

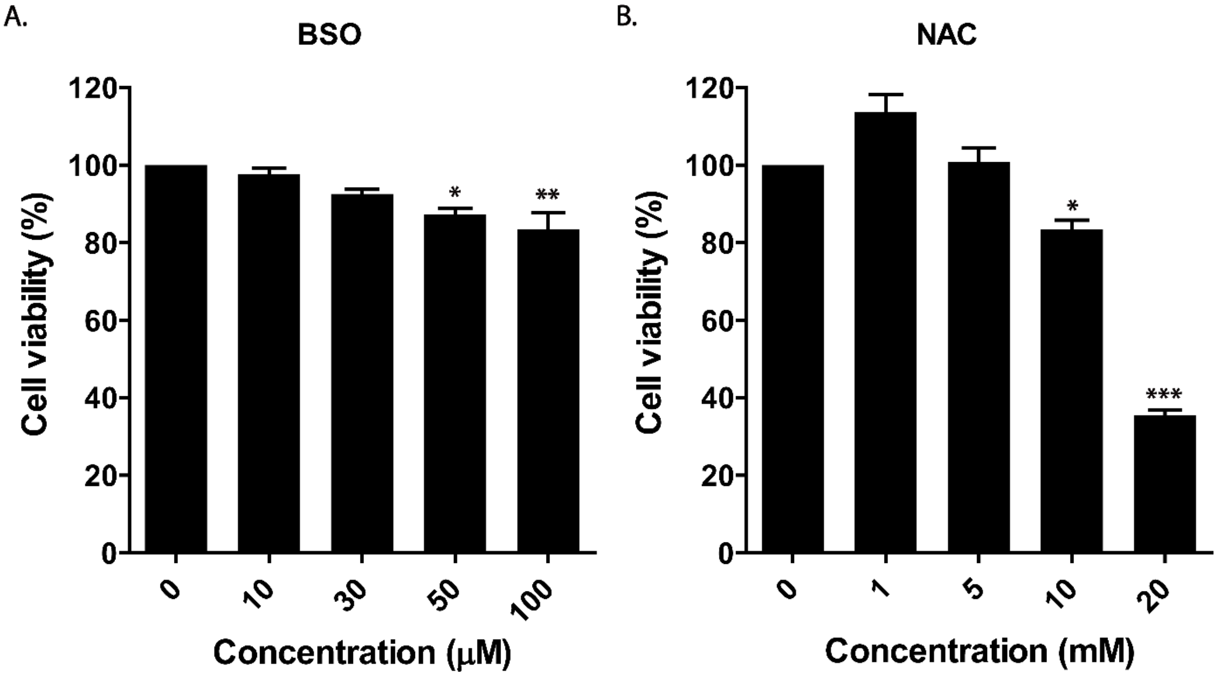

Figure S2. Viability of Nrf2 CALUX cells exposed to different concentrations of (A) BSO for 48 hours or (B) NAC for 28 hours. The data are presented as mean ± SEM of three independent replicates and * indicates a response significantly different from treatment of control (*, p<0.05; **, p<0.01; ***, p<0.001; one-way ANOVA analysis with post-hoc Tukey test).

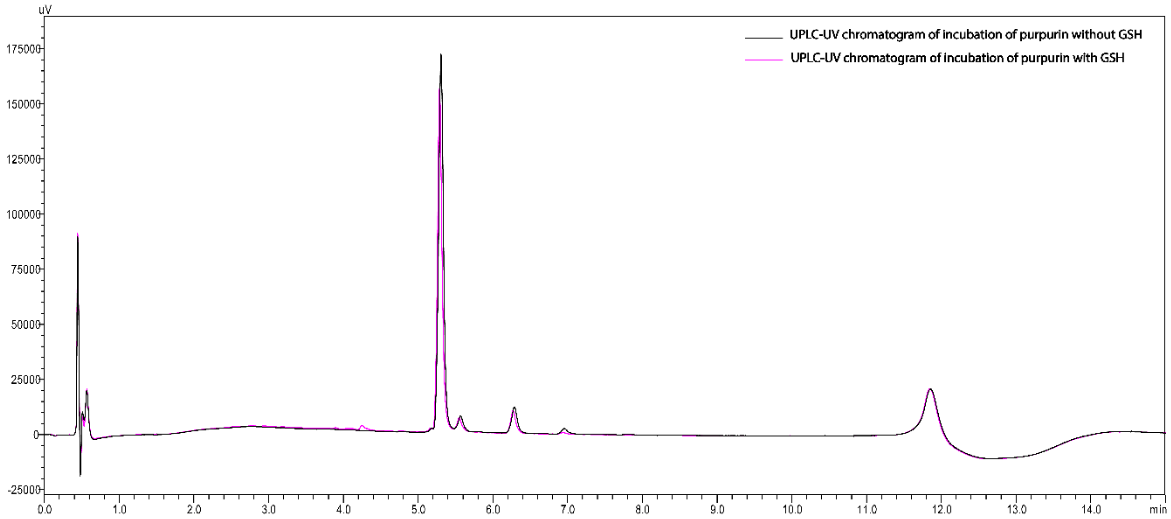

Figure S3. The comparison of the full views of HPLC-UV chromatograms of incubations of purpurin without (black line) with (pink line) GSH at pH 8.
